# Design, Fabrication, and Implementation of a Wireless, Passive Implantable Pressure Sensor Based on Magnetic Higher-Order Harmonic Fields

**DOI:** 10.3390/bios1040134

**Published:** 2011-10-17

**Authors:** Ee Lim Tan, Andrew J. DeRouin, Brandon D. Pereles, Keat Ghee Ong

**Affiliations:** Department of Biomedical Engineering, Michigan Technological University, 1400 Townsend Drive, Houghton, MI 49931, USA; E-Mails: eltan@mtu.edu (E.L.T.); ajderoui@mtu.edu (A.J.D.); bdperele@mtu.edu (B.D.P.)

**Keywords:** biomedical instruments, biosensors, higher-order harmonic fields, implantable sensors, magnetic detection, noninvasive monitoring, passive, pressure, soft magnetic materials, wireless sensors

## Abstract

A passive and wireless sensor was developed for monitoring pressure *in vivo*. Structurally, the pressure sensor, referred to as the magneto-harmonic pressure sensor, is an airtight chamber sealed with an elastic pressure membrane. A strip of magnetically-soft material is attached to the bottom of the chamber and a permanent magnet strip is embedded inside the membrane. Under the excitation of an externally applied AC magnetic field, the magnetically-soft strip produces a higher-order magnetic signature that can be remotely detected with an external receiving coil. As ambient pressure varies, the pressure membrane deflects, altering the separation distance between the magnetically-soft strip and the permanent magnet. This shifts the higher-order harmonic signal, allowing for detection of pressure change as a function of harmonic shifting. The wireless, passive nature of this sensor technology allows for continuous long-term pressure monitoring, particularly useful for biomedical applications such as monitoring pressure in aneurysm sac and sphincter of Oddi. In addition to demonstrating its pressure sensing capability, an animal model was used to investigate the efficacy and feasibility of the pressure sensor in a biological environment.

## 1. Introduction

One of the drawbacks of most current medical diagnosis systems is their inability to provide continuous long-term monitoring of physiological parameters. Although some *ex vivo* measurement devices such as the blood manometer are widely accepted and used in the medical community, they often fail to deliver reliable analytical results [1]. This leads to inaccuracy in measurements and, as a result, inappropriate disease treatment. Conversely, for patients who require full medical attention, percutaneous approaches, such as catheter-based pressure monitoring, are regularly used. Although this technique is more accurate than *ex vivo* measuring systems, issues such as pain, infection, and blood occlusion commonly occur. Therefore, under certain conditions, fully implantable sensors for physiological monitoring are perceived as an effective alternative for disease diagnosis as they can provide real-time, continuous, long-term monitoring in chronic patients.

One of the most common implantable sensors is the strain sensor, frequently employed in orthopedic research to assess bone morphology [2,3] and investigate bone diseases [4]. For example, an implantable sensor consisting of thin-film metal strain gauges embedded in a polydimethyl-siloxane membrane has been used to investigate osteoporosis and bone tumors. Another strain sensor, developed by MicroStrain Inc, uses piezoresistive strain gauges embedded inside a knee implant to measure bending, compressive, and shearing loads [5]. The sensor includes an embedded antenna and an implanted miniature inductive coil for wireless data transmission and power generation, respectively.

Pressure sensors are also used for *in vivo* assessment of implantable devices. Implantable wireless sensors have been used to provide continuous instantaneous monitoring of pressure in an abdominal aneurysm sac, following an endovascular aneurysm repair surgery, to detect the presence of endoleak. One such pressure transducer, the Remon Impressure AAA Sac pressure transducer, consists of a piezoelectric membrane that charges a capacitor with ultrasound waves, and then transmits the pressure measurement as an ultrasound signal remotely detected with a handheld probe [6]. Another sensor for this application, the EndoSure Wireless AAA Pressure Sensor manufactured by CardioMEMS Inc., monitors pressure with an inductive-capacitive resonance circuit that utilizes radiofrequency energy for power and data transmission [7]. However, the disadvantage of using radiofrequency energy is the large signal attenuation in electrically conductive human body compared to magnetic energy.

Aside from measuring strain and pressure, implantable sensors are also used for movement and flow monitoring. For instance, Rivero and coworkers presented a movement tracking sensor system for detecting heart valve bioprosthesis failure [8] by interrogating soft magnetic materials attached to the cusps of the heart valves. Bolz and coworkers designed an implantable flow sensor to improve the treatment of tachycardia, a form of cardiac arrhythmia, by measuring the presence of blood flow when using an implantable cardioverter defibrillator [9].

In this study, a wireless, passive pressure sensor based on the interaction of a magnetically-soft material and a permanent magnet was developed for biomedical application. Wireless, passive sensors making use of magnetic materials as the primary detection element have been previously reported for monitoring mercury vapor [10], strain or material deformation [11], fluid pressure [12,13], and ambient temperature [14]. The advantage of using magnetic materials in device fabrication is their passive nature which allows for permanent operation without an active power source. Additionally, their self-generation of magnetic fields allows for remote detection of the material, which is extremely advantageous in applications where frequent access to the device is difficult. Moreover, established microfabrication techniques provide for construction of miniature devices based on these magnetic materials with minimal design and fabrication complexities. Therefore, magnetic-based implantable devices designed for long-term, continuous monitoring are favorable as point-of-care diagnostic devices capable of improving patient care and treatment and, as a result, lowering healthcare cost.

The reported sensor, known as the magneto-harmonic pressure sensor, operates by exploiting the interaction of two ferromagnetic components: a magnetically-soft material and a magnetically hard material (permanent magnet). Under the excitation of an externally applied magnetic field, the magnetically-soft material with high magnetic permeability is capable of producing higher-order harmonic fields (magnetic fields at multiple frequencies of the excitation frequency). When exposed to an external DC magnetic field produced by a permanent magnet, the harmonic field is shifted, with the degree of harmonic shift depending upon the strength of the DC magnetic field. As a result, pressure measurement can be achieved by tracking the harmonic shift using a sensor in which the separation distance between the magnetically-soft material and the permanent magnet is dependent on the ambient pressure.

## 2. Theory

A theoretical model was developed to establish a relationship between the higher-order harmonic fields of the magneto-harmonic sensor and the magnetization (BH) curve of the material. Since measuring the BH curve is a widely adopted procedure for characterizing magnetic samples, the model can simplify the design, fabrication, and optimization process of the material when the ultimate goal is to realize a sample with a specific higher-order harmonic profile.

In previous work, it was shown that the higher-order harmonic pattern could be derived from the BH curve of the magnetically-soft material with a Fourier series transformation [14]. A piece-wise linear model was used to represent the BH curve so that the magnetization of the sample could be transformed from the time-domain magnetization curve to a frequency-domain amplitude spectrum. However, the model did not account for the BH curvature. A new theoretical model was developed to represent the BH curve of a magnetically-soft material. Assuming the material has a negligible hysteresis, the model represented the BH curve with exponential functions:

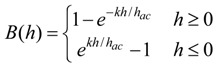
(1)
where *B* is the induced magnetic flux generated by the material, *h* is the applied field, *h_ac_* is the maximum amplitude of the applied AC field, and *k* is a constant describing the curvature of the BH curve. By applying the Fourier transform to the BH curve, the higher-order harmonic components of the BH curve can be determined as [14]:

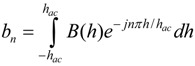
(2)
where *b* is the amplitude of the *n*^th^ harmonic field component. By substituting Equation (1) into Equation (2), the amplitude of the *n*^th^ harmonic field is:

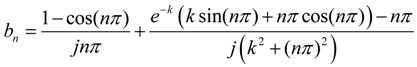
(3)


Equation (3) indicates that the amplitude of the *n^th^* order harmonic field can be determined from the material’s BH curve with given AC and DC excitation fields.

## 3. Experimental Section

### 3.1. Sensor Fabrication

#### 3.1.1. Electroplating of the Magnetically-Soft Material

Electroplated nickel-iron (NiFe) alloy films are a good candidate for the magnetically-soft materials due to their high magnetic permeability and low magnetization loss, in addition to being easy to fabricate. In the present work, a copper substrate (30 mm × 10 mm × 50 µm) was used as the cathode for the nickel-iron alloy deposition, while a nickel substrate used as the anode. These electrodes were immersed into a plating bath composed of 0.8 mol/L NiSO_4_·6H_2_O, 0.05 mol/L FeSO_4_·7H_2_O, 0.4 mol/L boric acid, 0.0164 mol/L saccharin, 0.1 mol/L sodium citrate dihydrate, and 0.005 mol/L sodium dodecyl sulfate [15]. The plating condition was maintained at temperature of 50 °C and pH level of 1.8. The plating bath was continuously stirred and the current density was maintained throughout the plating process at 16.7 mA/cm^2^.

#### 3.1.2. Electroplating of the Permanent Magnet

Magnetically-hard CoNiMnP films were electroplated and characterized. The experimental setup for the CoNiMnP electroplating process was similar to the NiFe fabrication process, except cobalt was used as the anode electrode instead of nickel. The chemical composition of the plating bath was as follows: 24 g/L CoCl_2_·6H_2_O, 24 g/L NiCl_2_·6H_2_O, 3.4 g/L MnSO_4_·6H_2_O, 4.4 g/L NaH_2_PO_2_·H_2_O, 25 g/L H_3_BO_3_, 24 g/L. NaCl, 0.18 g/L Sodium Saccharin, and 0.2 g/L Sodium Lauryl Sulfate [16]. The plating bath was maintained at room temperature and pH level of 3. The bath solution was agitated, and the current density was held at 6 mA/cm^2^.

#### 3.1.3. Sensor Body Fabrication

**Figure 1:**
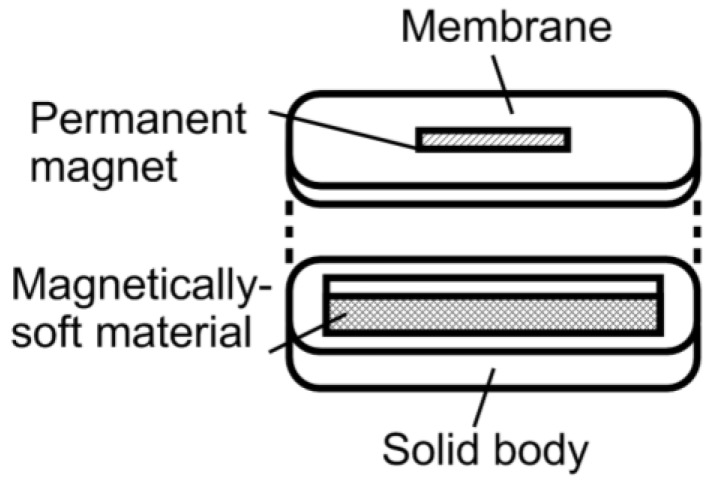
A diagram illustrating the design of the magneto-harmonic pressure sensor.

As illustrated in Figure 1, the magneto-harmonic pressure sensor consists of a sealed chamber with a flexible membrane adhered on the top. The sensor body was made of a rigid material to provide a strong structure, ensuring that only the membrane moved with changing pressure. Specifically, MACOR glass ceramic (manufactured by Corning Incorporated) was chosen as the rigid material due to its excellent material properties (zero porosity, non-shrinking, and hydrophobic). The pressure sensor body measured *L_1_* in length, *D_1_* in depth, and included an arch at both ends. At the center of the pressure sensor body was a rectangular well that measured *L_2_* in length, *w* in width, and *D_2_* in depth. Two sizes of sensor body were fabricated, known here as Sensor I and II. The dimensions of Sensor I are *L_1_* = 32 mm, *L_2_* = 28 mm, *D_1_* = 2.5 mm, *D_2_* = 2.175 mm, and *w* = 3 mm, while the dimensions of Sensor II are *L_1_* = 16 mm, *L_2_* = 13 mm, *D_1_* = 1.58 mm, *D_2_* = 1 mm, and *w* = 2 mm. Sensor I, with a larger sensor body, is more suitable for *in vivo* applications in a human, while the smaller Sensor II is designed for implantation in a small animal.

#### 3.1.4. Pressure Membrane Fabrication

To ensure biocompatibility [17] and sufficient membrane flexibility, a two-part medical grade silicone elastomer (Silastic^®^ MDX4-4210 BioMedical Grade Elastomer manufactured by Dow Corning Corporation) was used as the membrane material. Prior to casting the pressure membrane, a Teflon mold was first fabricated with a milling machine and, following this, the permanent magnet strip was positioned at the center of the Teflon mold. The two-part medical grade silicone elastomer (Silastic^®^ MDX4-4210 BioMedical Grade Elastomer manufactured by Dow Corning Corporation) was uniformly mixed and filled into the Teflon mold. Prior to curing, the mold was placed in a vacuum chamber at 648 mmHg in order to remove air bubbles resulting from mechanically stirring the elastomer. The elastomer was then cured in a vacuum oven at 100 °C for 15 min.

#### 3.1.5. Attachment of Magnetically-Soft Material and Permanent Magnet

The magnetically-soft material was housed within the sensor body to prevent any mechanical damage that would affect the efficacy of the pressure sensor. Commercial amorphous ferromagnetic material Fe_40_Ni_38_Mo_4_B_18_ (Metglas 2826MB manufactured by Metglas, Inc.) was used as the sensor component due to their consistent performance, allowing for rapid and large quantity sensor fabrication for testing purposes. To prepare the magnetically-soft strip, a purchased 2826MB reel (28 µm thick and 12.7 mm wide) was sheared into 28 mm × 3 mm and 13 mm × 2 mm for use in Sensor I and II, respectively. The sheared strips were then attached to the bottom of the sensor body. Similarly, commercial permanent magnet strips were also used to ensure that a large quantity of sensors could be fabricated for testing purposes. Specifically, commercial Fe_60_Cr_30_Co_10_ (Arnokrome^TM^ III manufactured by Arnold Magnetic Technologies Corp.) was selected for this project as its magnetic and mechanical properties match the desired sensor specifications. To prepare the permanent magnet strip, Arnokrome^TM^ III with thickness of 40 µm was sheared into 21 mm × 2 mm and 1.27 mm × 10.16 mm strips for use in Sensor I and II, respectively. Note that the electroplated magnetically-soft and hard materials descried earlier were only used to investigate the feasibility of customizing and fabricating magnetic materials with unique magnetic properties as a possibility to suit specific applications. To maintain performance consistency, the sensors were made with commercial magnetic materials, which have similar properties with the fabricated materials (see Results and Discussion section).

#### 3.1.6. Sensor Body-Membrane Interface Adhesive

Silicone adhesive (Silbione^®^ MED ADH 4100 RTV manufactured by Bluestar Silicones) was used to seal the sensor body to the pressure membrane. Adhesive was applied on the edges (width of 1.5 mm) of the sensor body allowing for permanent attachment of the pressure membrane to the sensor. To ensure complete contact between the membrane and sensor, the pressure membrane was gently pressed against the adhesive covered edges. The completed sensor was then cured at room temperature (25 °C) for 72 h.

### 3.2. Higher-Order Harmonic Excitation and Detection System

**Figure 2:**
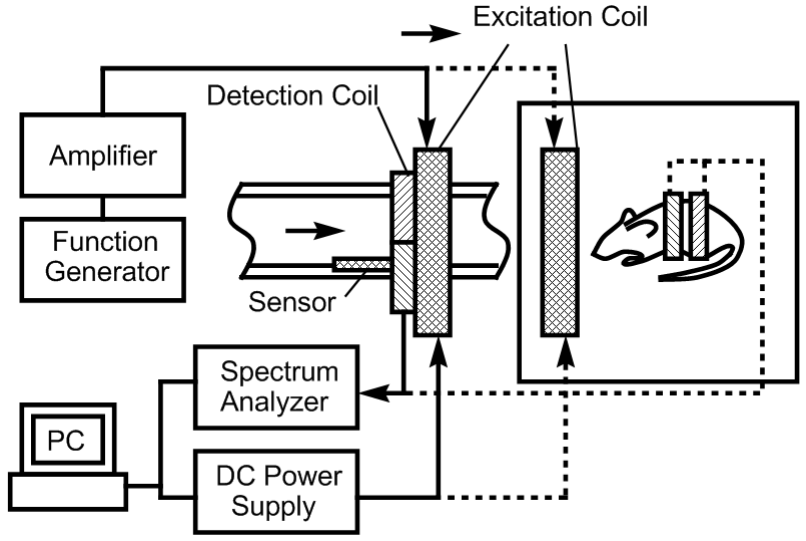
Experimental setup for the sensor evaluation and characterization.

The pressure sensor’s excitation coil was consisted of a two-superimposed magnetic coil (AC and DC coils). A function generator (Fluke 271 10 MHz) and an amplifier (Tapco 1400) were used to generate an AC current to the AC coil. The DC coil obtained its input signal from a DC power supply (Kepco BOP 30-10) controlled by a computer interface. Once the pressure sensor was activated, the generated signal was detected with a receiving coil and processed with a spectrum analyzer (Agilent 4396A). A customized Microsoft Visual Basic 6 program was used to control the measurement process and communicate with the devices via IEEE 488.2 interface. Figure 2 illustrates the electronic instruments involved in the system.

The excitation coil was consisted of a circular coil of 26 cm in diameter made by winding 63 turns of 18-gauge magnet wire. The receiving coil (co-planar with the excitation coil) was composed of two circular coils, each consisting of 200 turns of 18-gauge magnet wire a diameter of 2.5 cm. To prevent capturing the excitation fields, the two coils were connected in series but in opposing winding directions. As a result, the induced signal from the output of the receiving coil was solely generated by the pressure sensor. Although the current setup can monitor up to the 6th harmonic fields, only the 2nd-order harmonic field was used due to the higher signal-to-noise ratio. 

Figure 2 also illustrates the experimental setup used to evaluate and characterize the performance of the magneto-harmonic pressure sensor. For the *in vitro* experiment, the sensor was embedded within a conduit wall and was placed in close proximity to the excitation and receiving coils. Fluid flow was controlled using a flow/pressure regulator and the conduit pressure was measured with a commercial pressure meter. A 200 Hz sinusoidal signal, produced by the function generator, remotely excited the pressure sensor, which was then exposed to changing fluid pressures. At each increment of fluid pressure, the 2nd-order harmonic field was remotely acquired by the receiving coil connected to the spectrum analyzer. For the *in vivo* experiment, a figure-8 receiving coil, worn over the torso of the mouse, was placed at the center of the AC-DC excitation coil. The pressure sensor was excited with a 5.88 × 10^−4^ A/m magnetic field at 200 Hz while the 2nd-order harmonic field was measured.

## 4. Results and Discussion

### 4.1. Characterization of the Electroplated Magnetically-Soft Material

The magnetic properties of electroplated nickel-iron alloys were characterized in terms of saturation magnetization (*M_s_*), coercivity (*H_c_*), and remanence (*M_r_*) through the BH loops, measured with an alternating gradient magnetometer (AGM) (MicroMag^TM^ Model 2900 by Princeton Measurements Corporation). The samples were cut into 4 mm × 4 mm pieces and attached to the carrier of the AGM probe.

**Figure 3:**
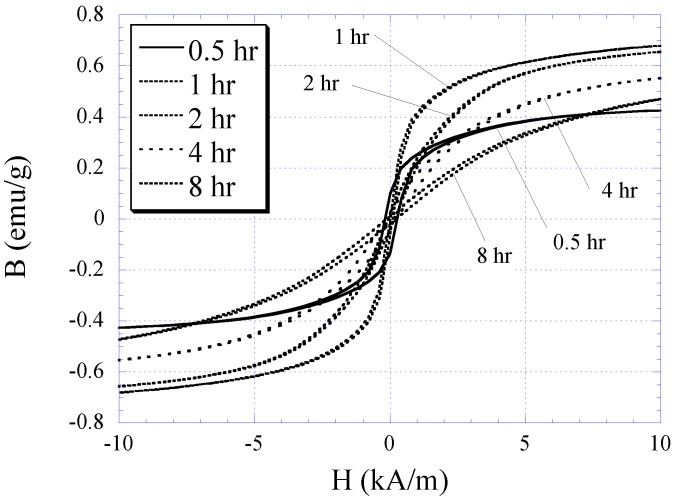
The BH responses for 0.5, 1, 2, 4, and 8 h electroplated nickel-iron samples.

**Figure 4:**
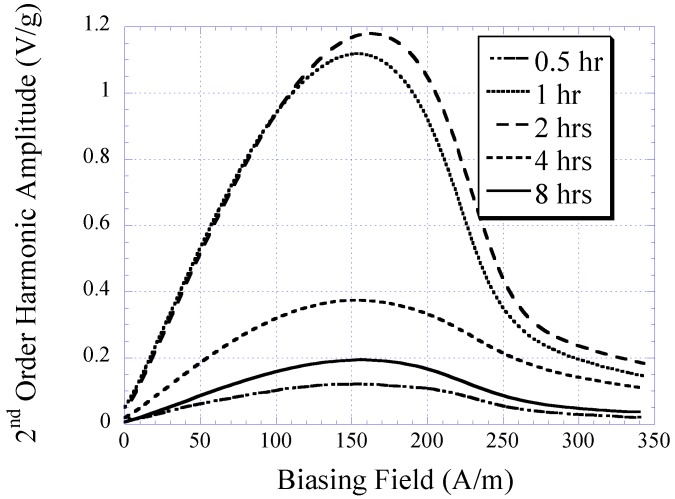
The 2nd-order harmonic fields exhibited by electroplated nickel-iron alloys plated for 0.5, 1, 2, 4, and 8 h normalized to the sample mass.

**Figure 5:**
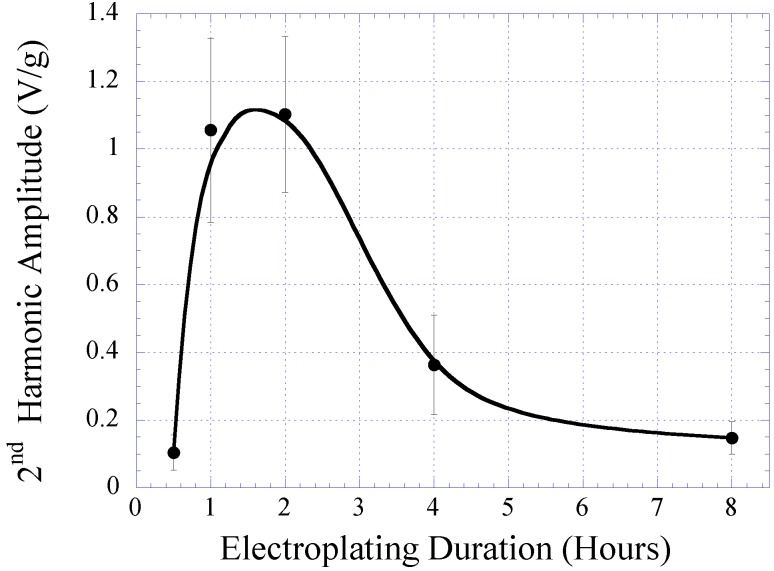
The maximum amplitude of the 2nd-order harmonic fields exhibited by electroplated nickel-iron alloys plated for 0.5, 1, 2, 4, and 8 h normalized to the sample mass.

The characteristic of electroplated nickel-iron alloy as a function of plating duration was also investigated. Copper substrates were separately electroplated with plating durations of 0.5, 1, 2, 4, and 8 h while maintaining the condition of other plating parameters (*i.e*., current density, temperature, and pH) throughout. The BH responses of these samples are plotted in Figure 3 with the saturation magnetization *M_s_*, coercivity *H_c_*, and anisotropy field *H_k_* listed in Table 1. As shown in the table, the *M_s_* and *H_k_* of the samples increased with plating duration up to 2 h, and then reduced. Conversely, *H_c_* demonstrated an initial decrease and then increased with higher plating time. It is believed that the initial increase in the soft ferromagnetic behavior at low plating time was due to the formation of a thicker, more uniform coating. However, the reduction of the soft magnetic behavior at thicker samples could be explained by the increase in internal stress within the thick layers, which led to an increase in magnetic coercivity. Since the higher-order response of a material is a direct reflection of the permeability and coercivity of the sample, the same pattern was observed in the higher-order response *versus* plating duration (see Figure 4 and Figure 5). Figure 4 indicates the 2nd-order harmonic fields generated by the electroplated soft magnetic materials are equivalent to those generated by commercial soft magnetic materials such as Metglas 2826MB (the 2nd-order harmonic response of Metglas 2826MB was described in the previous work [11,12]).

**Table 1:** The saturation magnetization, anisotropy field, and coercive force of nickel-iron samples of different plating duration.

Electroplating Duration (h)	Saturation Magnetization *M_s_* (emu/g)	Anisotropy Field *H_k_* (kA/m)	Coercive Force *H_c_* (A/m)
0.5	0.455	0.75	440
1.0	0.726	1.52	80
2.0	0.705	2.75	216
4.0	0.617	4.23	384
8.0	0.580	6.62	328

**Figure 6:**
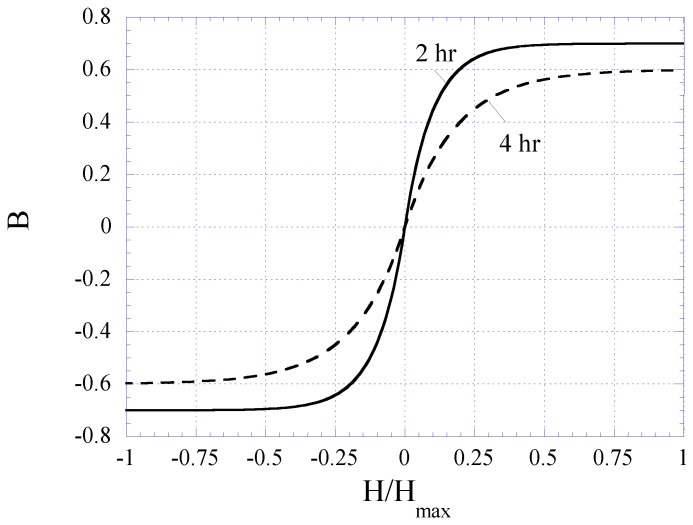
Exponential functions best-fitted to the BH loops of the 2 and 4 h plated nickel-iron alloy samples.

**Figure 7:**
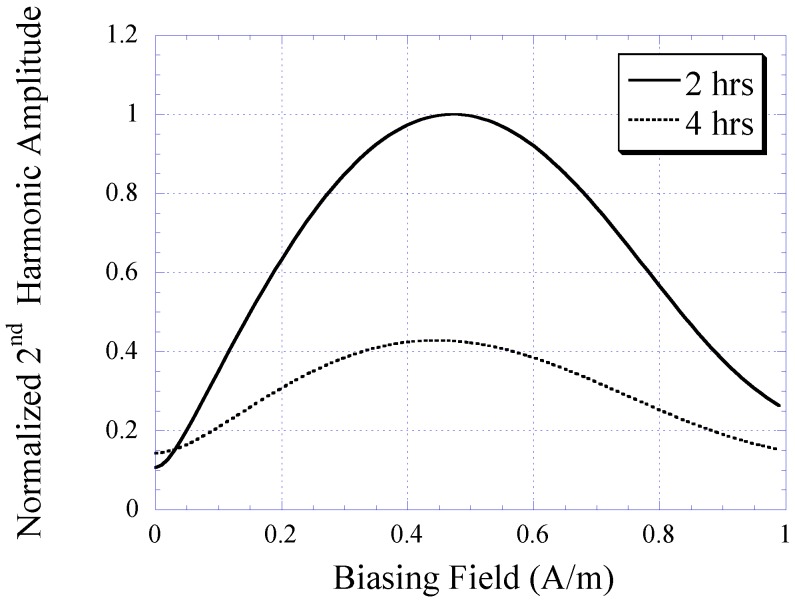
The 2nd-order harmonic fields of nickel-iron alloy samples (2 and 4 h plated) determined with the theoretical model.

Figure 6 plots the BH curves modeled with Equation (1). To fit to the measured BH curves of 2 and 4 h plated samples, the value of *k* in Equation (1) was determined as 12 and 6 for 2 and 4 h samples, respectively. The 2 and 4 h curves were also normalized by a factor of 0.7 and 0.6, respectively. The 2nd-order harmonic fields of the 2 and 4 h samples were determined using Equation (3) and plotted in Figure 7. Although the modeled 2nd-order harmonic fields showed the same trend as the measured 2nd-order harmonic fields, there were observable differences caused by variations between the actual and modeled curvatures of the BH plots. Nevertheless, the model provided a simple technique to predict the higher-order fields of soft magnetic materials based on their BH curves.

### 4.2. Characterization of the Electroplated Permanent Magnet

Electroplating of CoNiMnP on copper substrates was conducted at durations of 1, 4, and 8 h. Using a gaussmeter (DC Gaussmeter Model GM2 by AlphaLab, Inc.), the maximum magnetic induction produced by the electroplated CoNiMnP was obtained and listed in Table 2. As shown, when the plating duration increased, the magnetic induction generated by CoNiMnP increased. It is known that the magnetic induction of electroplated magnetic materials is a function of deposition thickness or mass, and the deposition thickness is a function of deposition current and duration as described in Faraday’s law, *m* = *αtIM*/*nF*, where *m* is the mass of deposited materials, *α* is the current efficiency, *I* is the applied current, *t* is the deposition time, *n* is the charge of the deposited ions, *M* is the mass of deposited materials, and *F* is the Faraday’s constant [18]. It was also reported in other works that an increase of deposition thickness resulted in higher magnetic energy stored in magnetic material [16]. Hence, when the plating duration was prolonged, thicker deposition of CoNiMnP was formed on the copper substrate, resulting in greater magnetization.

**Table 2:** The magnetic induction generated by electroplated CoNiMnP plated for 1, 4, and 8 h.

CoNiMnP Plating Duration (h)	Magnetic Induction (T)
1	2 × 10^−3^
4	6 × 10^−3^
8	8.5 × 10^−3^

**Figure 8:**
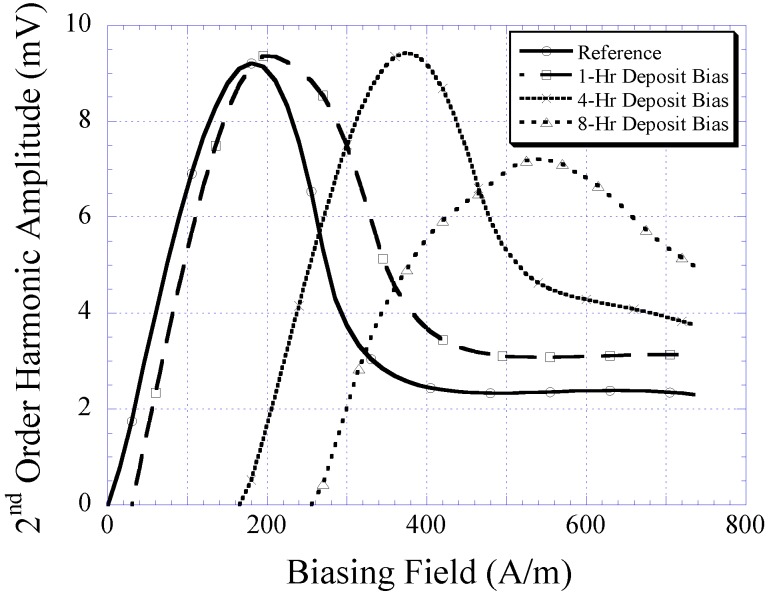
The 2nd-order harmonic shift produced by magnetically-soft material in the presence of 1, 4, and 8 h electroplated CoNiMnP samples.

Besides using the gaussmeter, the magnetic properties of the electroplated CoNiMnP were evaluated in terms of its ability to shift the higher-order harmonic field of a magnetically-soft material. Shown in Figure 8 is the 2nd-order harmonic field produced by the magnetically-soft material that was magnetically biased with CoNiMnP samples electroplated at 1, 4, and 8 h. The reference curve indicates the 2nd-order harmonic field in the absence of the DC magnetic biasing field. As shown, when the magnetically-soft material was exposed to a DC magnetic biasing field, the 2nd-order harmonic field was shifted rightward, with 8-h electroplated CoNiMnP showing the greatest shift followed by the 4 and 1 h electroplated CoNiMnP samples. Compared to the commercial magnet strip Arnokrome III, the electroplated permanent magnet strip provided a similar biased harmonic spectrum for a soft magnetic material (the biased harmonic spectrum by Arnokrome III strips was described in the previous work [11,12]).

### 4.3. *In Vitro* Evaluation of the Sensor Performance

**Figure 9:**
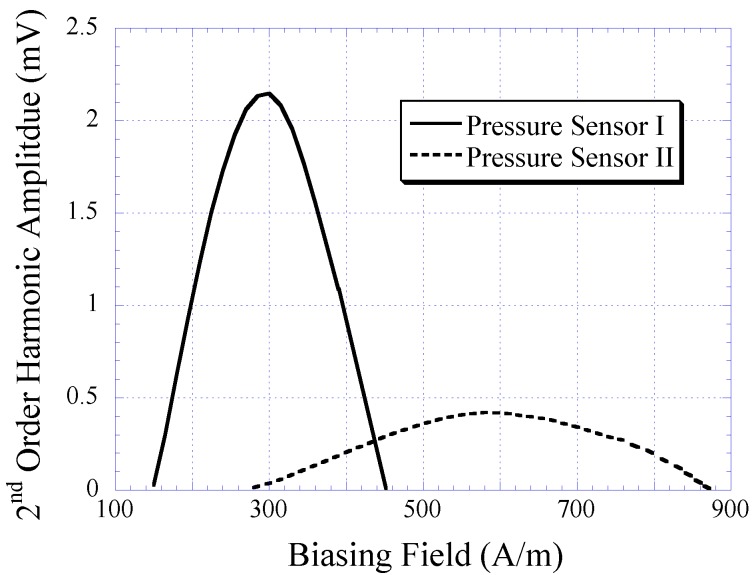
The 2nd-order harmonic fields produced by Sensors I and II. Note that the harmonic shift presented was due to the variation in separation distance between the magnetically-soft material and the permanent magnet, which was different for Sensors I and II.

The 2nd-order harmonic fields produced by Sensor I and II are illustrated in Figure 9. As shown, the 2nd-order harmonic amplitude produced by Sensor I was significantly higher than that of Sensor II. An amplitude strength reduction of approximately 80% was seen when the size of the magnetically-soft magnetic strip was reduced to approximately 50%. Although the amplitude of the harmonic field is not the focus of this study, its magnitude represents the signal strength of the pressure sensor. High signal strength is crucial to maintain a high signal-to-noise ratio and ensure the reliability of signal measurements. Note that the harmonic amplitude did not affect the 2nd-order harmonic shift. The harmonic shift shown in Figure 9 was caused by the separation distance between the magnetically-soft material and the permanent magnet, which was different for Sensor I and II. Sensor I and II consisted of a well with a depth of 2.175 mm, and 1.5 mm, respectively. When the separation distance decreased, the exposure of DC magnetic biasing field on magnetically-soft material increased. As a result, a larger shift of the 2nd-order harmonic field occurred.

**Figure 10:**
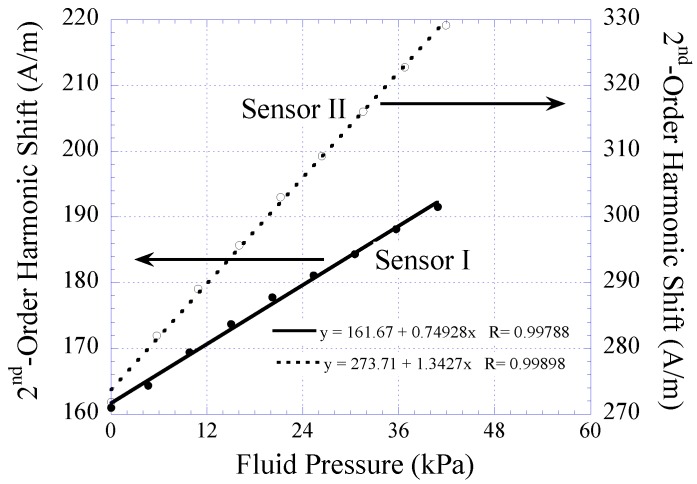
The 2nd-order harmonic shift obtained from Sensor I and II when exposed to varying fluid pressure. Both the sensors exhibited linear signal response with detection sensitivity of 0.749 and 1.34 A/m∙kPa, respectively.

The harmonic shift exhibited by Sensors I and II when exposed to fluid pressure ranging 2 to 44 kPa was also measured. When the fluid pressure was increased, the sensor membrane, embedded with permanent magnet, was deflected toward the magnetically-soft material. This caused the biasing field exposed to the magnetically-soft element to increase. As a result, a greater shift of the 2nd-order harmonic field was observed. As shown in Figure 10, the harmonic shift was found to increase linearly as a function of increasing fluid pressure. The sensor’s sensitivity, which was determined by the slope of the individual curve, was found to be 0.749 A/m·kPa for Sensor I and 1.34 A/m·kPa for Sensor II.

**Figure 11:**
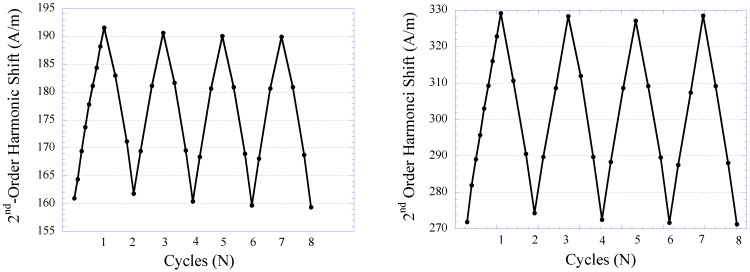
The repeatability of the 2nd-order harmonic shift produced by Sensor I (**left**) and Sensor II (**right**).

The pressure sensor’s repeatability under several cycles of varying fluid pressure environment was also investigated. Similar to the technique described earlier, the fluid pressure was increased from 2 to 44 kPa and at each predetermined pressure value the response of the pressure sensor was acquired. The fluid pressure was then reduced in a similar trend in the opposite direction down to 2 kPa and, again, at each pressure value the sensor’s response was acquired. Shown in (Figure 11(a,b)) are the repeatability exhibited by Sensor I and II, respectively. As shown, both Sensor I and II exhibited good repeatability when tested for eight cycles of pressure-changing environment. Noticeable drift was observed, particularly in the first few cycles, and subsequently stabilized when approaching the end cycle. Maximum drift on the lowest detection limit exhibited by Sensor I and II were 0.87%, and 0.9%, respectively. Maximum drift on the highest detection limit for Sensor I and II were 0.48%, and 0.42%, respectively. Although the drift displayed by Sensor I and II are considered insignificant, in practice the pressure sensor can be preloaded in a pressure-changing environment before sensor implantation to improve the stability of sensor performance.

### 4.4. *In Vivo* Evaluation of the Sensor Performance

Sensor II was implanted into a mouse to evaluate its performance *in vivo*. BALB/c mice were chosen as the animal model because they provide a simple reproducible model for determining the host response to an implanted composite biomaterial and were used in animal models for similar studies [19,20,21]. Eight male BALB/c mice of age 3 to 4 weeks were housed in a specific-pathogen-free (SPF) animal facility. Subcutaneous sensor implantation was conducted using previously established implantation techniques [19]. BALB/c mice were anesthetized with an isofluorane-oxygen gas mixture followed by hair removal and antiseptic application around the incision sites to reduce the possibility of infection. Using surgical instruments sterilized with 100% ethyl alcohol (EtOH), a small horizontal incision at the mid-lower dorsal of the mouse was made. The subcutaneous tissue was exposed with the horizontal incision that allowed for the following vertical cut along the spine of the mouse. Then, a pouch at the left dorsal of the mouse was made by horizontally sliding a closed scissors towards the down side of the dorsal followed by opening the scissors in order to create a pocket for sensor implantation. After fixation of the sensor, the dorsal incision wound was closed using 2 surgical-grade stainless steel staples and the mouse was allowed to recover.

**Figure 12:**
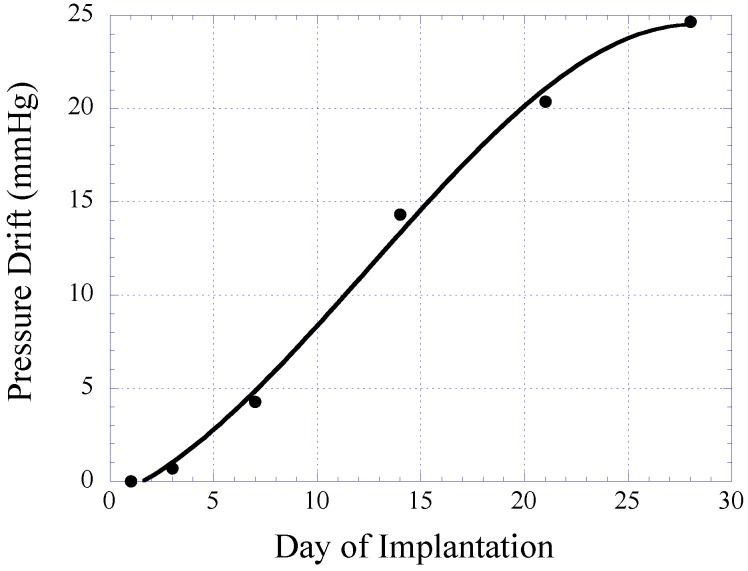
Plot illustrating the sensor drift of the magneto-harmonic pressure sensor in 28 days of implantation. The plot is generated using data points obtained from 14- and 28-day study groups.

In this study, 14- and 28-day implantations were chosen as the time points. For the 14-day implantation, the response of the pressure sensor was evaluated at day-1, 3, 7, and 14 for signs of sensor signal drift. For the 28-day implantation, the response of the pressure sensor was evaluated at day-7, 14, 21, and 28. Figure 12 illustrates the drift in sensor response obtained from the 28-day implanted pressure sensor. Given the pressure range of the sensor, the resulting sensor drift is considered small. Furthermore, it was demonstrated that the sensor drift became stabilized towards the 28-day of implantation. Thus, it is believed that the accuracy of pressure detection will improve after 1 month sensor implantation.

**Figure 13:**
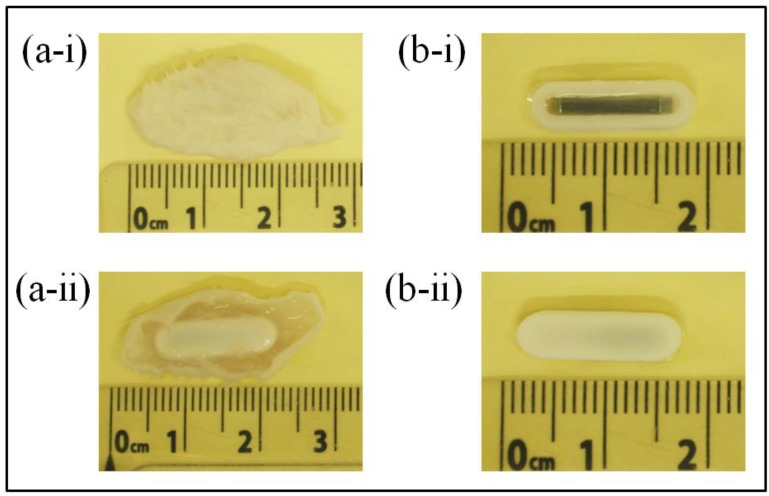
Photographs showing the (**a**) sensor embedded in skin-tissue structure and (**b**) 28-day implanted sensor after skin removal. ‘**i**’ and ‘**ii**’ indicate the front and back of the surface of the sensor respectively.

At the end of each experimental group (14- and 28-day sensor implantations), BALB/c mice were sacrificed using CO_2_ asphyxiation technique approved by the American Veterinary Medical Association (AVMA). A mixture of O_2_ and CO_2_ gas was gradually introduced to the mice at ratios of 0.75:0.25, 0.5:0.5, 0.25:0.75, and 0:1 with 5 min of exposure at each mixture of gas. After confirmation of successful euthanasia, sensor explantation was performed by removing the fur at the explantation site followed by cutting the skin and tissue (skin-tissue structure) around the sensor. Photographs of the skin-tissue embedded sensor and skin-removed sensor are shown in Figure 13. Qualitative assessment of 28-day implanted and non-implanted sensors demonstrated minimal tissue/fibrous formation over the surface of the sensor. The lack of extensive tissue formation on the sensor surface is consistent with the results in Figure 12, which indicates a minimal drift in harmonic shift during 28 days of implantation.

Further performance evaluation and characterization was also conducted on sensors that were explanted from the skin-tissue structure. The explanted sensors were inserted into a flow conduit regulated by a flow regulator. The 2nd-order harmonic shift produced by 14- and 28-day implanted sensors was measured as a function of fluid pressure and is listed in Table 3. The harmonic shift exhibited by a non-implanted pressure sensor, referred here as the reference sensor, was used for comparison of implanted and non-implanted sensor performance. As shown, both 14- and 28-day implanted sensors exhibited higher detection sensitivity as compared to the reference sensor. It is believed that cell/tissue contraction around the body of the sensor created a pre-stressed condition at the sensor membrane, resulting in greater detection sensitivity. As shown in the graph, a 28-day implanted sensor exhibited higher detection sensitivity as compared to a 14-day implanted sensor. This outcome was expected due to the possible greater cell contraction during the 28-day sensor implantation.

**Table 3:** The pressure response for the reference and implanted sensor at 14-day and 28-day implantation time.

Sensor	Harmonic Shift at 40 kPa
Control	42 A/m
14 Day Implantation	63 A/m
28 Day Implantation	69 A/m

**Figure 14:**
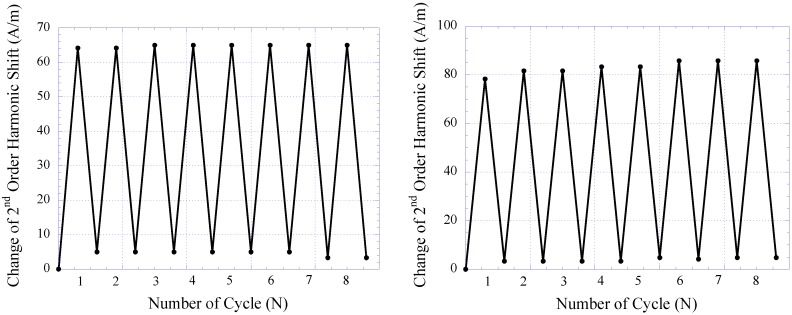
The repeatability of 14-day (**left**) and 28-day (**right**) implanted magneto-harmonic pressure sensors.

The repeatability of the pressure sensor embedded within the skin-tissue structure was also evaluated and characterized. Here, the sensor was continuously loaded in a pressure chamber at 2 and 44 kPa for 8 loading cycles. At each pressure point, the 2nd-order harmonic signal produced by the sensor was measured. Figure 14 plots sensor response during repeatability testing of the 14- and 28-day implanted pressure sensors. As shown, both 14- and 28-day implanted sensors exhibited good repeatability with minimal performance drift. For 14- and 28-day implanted sensors, the greatest drift occurred at the 1st and 2nd cycles of pressure loading, which contributed about 8 and 4.25% of full-span output of the sensor, respectively. It was also shown that after the 2nd cycle of pressure loading, both 14- and 28-day implanted sensors demonstrated greater stability. This finding is consistent with the results obtained from *in vitro* sensor testing.

For the tissue histology examination, the surrounding tissue capsule from the 28-day implanted sensor was fixed with methyl Carnoy’s and embedded in optimum cutting temperature (OCT) compound in a vacuum at 648 mmHg (25.5 inch Hg) for 12 h to remove air bubbles and allow for the penetration of the OCT compound into the tissue matrix. The embedded tissue capsule was then allowed cure at low temperature followed by frozen sectioning with a cryomicrotome (Microtome HM 550) at a sectioning thickness of 35 µm. The sliced tissue was subsequently immersed in water to remove the OCT compound from the tissue. Tissue was later retrieved from the water and dehydrated via alcohol for subsequent histological examination.

In this study, hematoxylin and eosin (H&E) staining was used to make the initial assessment of the *in vivo* host response to the magneto-harmonic pressure sensor. H&E staining is used to determine the spatial distribution of cellular and fiber matrix components local to the implantation site. Briefly, sectioned tissue was immersed in hematoxylin (Gill 3) for 10 s, followed by rinsing in water for 5 min, and 5 washes in 95% alcohol. To counterstain the tissue, eosin Y prepared with 24.87% (by volume) of eosin Y stock solution, 74.62% of 80% alcohol, and 0.5% of concentrated glacial acetic acid was added to the tissue for 30 s. Then, tissue was dehydrated using 95% alcohol and absolute alcohol for 5 min each. Lastly, the dehydrated tissue was cleared in two changes of xylene for 5 min each. Using this staining process, cell nuclei would appear purple and fiber matrix would appear pink under light microscopy.

**Figure 15:**
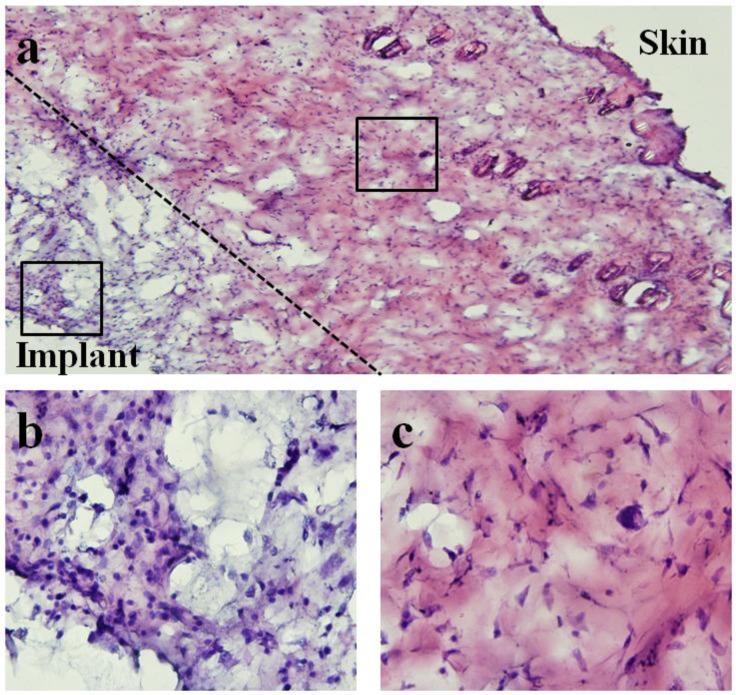
Light microscope images of hematoxylin and eosin (H&E)-staining of surrounding tissue capsule from 28-day implanted pressure sensor. Note that for histological examination purposes, the soft tissue capsule was removed from the pressure sensor prior to embedding and sectioning. (**a**) 10× image of both skin and implant sides of surrounding tissue capsule. (**b**,**c**) 60× images of implant and skin sides respectively. Differences in cellularity are consistent with qualitative assessment where minimal chronic and fibrotic inflammatory response was verified at time of explantation.

Figure 15 shows the microscope image of H&E stained tissue taken with an Olympus BX51 microscope. An image at 10× magnification was obtained to demonstrate both skin and implant sides of the surrounding tissue. Higher magnification images (60×) for different regions of tissue were also obtained to show the spatial distribution of cellular and fiber matrix components. As shown, tissue closer to the implant side consisted of a higher concentration of cells and lower concentration of fiber matrix. In contrast, tissue closer to the skin side contained a significantly higher concentration of fiber matrix.

Qualitatively, differences in cellularity and fiber matrix components at the implant and skin sides were consistent with the assessment during time of explantation where minimal chronic and fibrotic inflammatory response around the sensor was observed (see Figure 12 and Figure 13). The finding is consistent with previous studies where implantation of similar materials demonstrated persistent chronic inflammation characteristic with the accumulation of leukocytes after 45 days of implantation [22]. However, some studies have suggested that the accumulation of leukocytes may be the product of implant contamination [23,24] and part of wound healing following the surgical intervention. Further examinations are needed to determine the type of cell located at the implant side of the tissue. Nevertheless, histological examination has revealed that sensors implanted for 28 days promoted minimal fibrotic encapsulation, which is particularly crucial for the functionality of the sensor.

## 5. Conclusions

The efficacy and feasibility of a wireless passive pressure sensor system, known as the magneto-harmonic pressure sensor, was presented. An improved theoretical model was also developed for the generation of higher-order harmonics by magnetically-soft materials. Using this theoretical model, the characteristic of the harmonic field at specific frequencies was determined with ease. In addition, an excitation and receiving system was designed and fabricated for the excitation and detection of the pressure sensor. Furthermore, electroplating of the magnetically-soft material and the permanent magnet strip was described. The pressure sensor was tested in both *in vitro* and *in vivo* experiments and demonstrated responses proportional to the ambient pressure in addition to maintaining functionality for up to 28 days of animal implantation.

Although the efficacy and feasibility of this sensor technology was demonstrated, future work can be done to enhance the design and performance of the pressure sensor system. Primarily, the excitation and receiving system currently uses commercially available electronic instruments to generate the excitation field and process the magnetic response generated by the sensor. Although this worked well in a laboratory setting, it is not feasible for a portable/wearable system. In the future, a miniature excitation and receiving system will be developed to allow for greater mobility.

Aside from improving the excitation and receiving system, the evaluation of a magneto-harmonic pressure sensor in an animal model can be performed for an extended time period to investigate the functionality and durability of pressure sensor for long-term implantation. To accomplish this task, the pressure sensor should be implanted in the animal body for a longer duration. During the period of implantation, the functionality of the implanted pressure sensor will be evaluated to prove the feasibility of long-term implantation and, at the end of implantation, the pressure sensor will be retrieved and histological studies will be performed to determine the degree of host response to the sensor.
